# Assessment of Corneal Graft Outcomes in a Murine Model of Endothelial Keratoplasty

**DOI:** 10.3390/jcm13175010

**Published:** 2024-08-24

**Authors:** Akitomo Narimatsu, Rohan Bir Singh, Pier Luigi Surico, Seokjoo Lee, Katayoon Forouzanfar, Francesca Kahale, Aytan Musayeva, Thomas H. Dohlman, Tomas Blanco, Reza Dana

**Affiliations:** Laboratory of Ocular Immunology, Transplantation and Regeneration, Schepens Eye Research Institute, Massachusetts Eye and Ear, Department of Ophthalmology, Harvard Medical School, Boston, MA 02114, USA; anarimatsu@meei.harvard.edu (A.N.); rohan_singh@meei.harvard.edu (R.B.S.); psurico@meei.harvard.edu (P.L.S.); seokjoo_lee@meei.harvard.edu (S.L.); kforouzanfar@meei.harvard.edu (K.F.); francesca_kahale@meei.harvard.edu (F.K.); aytan_musayeva@meei.harvard.edu (A.M.); thomas_dohlman@meei.harvard.edu (T.H.D.); tomas_blanco@meei.harvard.edu (T.B.)

**Keywords:** endothelial keratoplasty, murine model, graft failure, graft rejection

## Abstract

**Objectives**: In this study, we establish a protocol for evaluating the outcomes of endothelial keratoplasty, including graft survival, rejection, or failure. Additionally, we also evaluate the alloimmune response in graft recipients. **Methods**: We performed EK using C57BL/6 (allogeneic) and BALB/c (syngeneic) as donors and BALB/c mice as recipients. Slit-lamp examination and optical coherence tomography were performed for clinical evaluations for 16 weeks post-procedure. Criteria for the assessment of corneal opacity were established and the animals were graded weekly. Additionally, we assessed corneal endothelial cell density by harvesting the corneas and staining with zonula occludens-1 (ZO-1). Lastly, lymph nodes were collected, and CD4^+^ T cells were MACS-sorted and co-cultured with syngeneic or allogeneic antigen-presenting cells (APCs) to assess the IFN-γ expression levels by alloreactive Th1 cells (ELISPOT) in response to the direct (donor) or indirect (host) pathways of sensitization. **Results**: We observed graft failure in four animals, including irreversible corneal opacity, graft detachment, and anterior synechiae in the first four weeks. The remaining animals were graded between 0 and 5 as per the established criteria. The total and graft corneal thickness and endothelial cell density progressively worsened with a higher grade of corneal opacity. The direct allosensitization of Th1 cells was significantly higher in mice with a higher grade of corneal opacity. At 16 weeks follow-up, the grafts remained stable with low opacity scores in syngeneic EK recipients; however, the opacity scores were higher and variable in allogeneic EK recipients. **Conclusions**: These findings establish a standardized protocol to assess the graft outcomes in a murine model of EK. Furthermore, we delineate the underlying immunological pathway that contributes to the immune-mediated rejection of grafts in this model.

## 1. Introduction

Corneal transplantation is one of the most commonly performed solid tissue transplantation procedures globally [1]. Despite the high success rates of penetrating keratoplasty (PK), the procedure is associated with increased risk of graft rejection, especially in high-risk settings such as previous graft failures, repeated rejections, host bed inflammation, a history of ophthalmic surgeries and trauma, and donor-related factors such as diabetes and dry eye [2,3,4,5]. Moreover, PK is associated with various post-procedure complications such as suture related graft infection, graft detachment, and glaucoma [6,7,8]. In contrast, endothelial keratoplasty (EK) is a less invasive procedure and requires smaller incisions, thus mitigating the risk of complications such as wound dehiscence, surgically induced astigmatism, hemorrhage, iridocorneal synechiae, and infections [9,10,11]. Additionally, there is no procedure-related corneal nerve damage and loss of sensation, the ocular surface integrity is maintained, and there is notably lower immune-mediated graft rejection in EK compared to PK [12,13]. Hence, EK has emerged as the primary form of keratoplasty in the United States for the treatment of endothelial dystrophies, which are one of the common corneal disorders causing vision loss [14,15]. Despite the wide adoption of EK due to its well-established advantages over PK, there is a gap in the knowledge pertaining to the underlying mechanisms that cause graft failure in EK [13]. In human studies, 8–14% endothelial immune rejection has been reported post-DSAEK, compared to 5–17% post-low-risk PK. [16,17,18,19,20] Several studies have reported immune-mediated rejection between 2.2 and 5.3% post-DMEK [21,22,23]. The lower rates of immune-mediated graft rejection in EK can be attributed to the reduced exposure of the endothelial graft to APCs in the recipient cornea, which are primarily located in the anterior stromal layer of the tissue. Moreover, significantly fewer APCs that also contribute to graft rejection accompany the grafted tissue in EK. Another important factor is the absence of corneal sutures, which may become loose in cases of PK and induce inflammation and a secondary immune reaction.

Despite the advantages of EK over PK, immune-mediated graft rejection is observed post-procedure. Therefore, we established a murine model to evaluate these underlying changes following this procedure [24]. However, it is essential to develop a standardized protocol for the clinical assessment of the corneal endothelial grafts transplanted in the recipient animals to ensure consistency and the reproducibility of the outcomes [25,26,27]. Additionally, it is necessary to delineate the immune-mediated mechanisms underlying graft rejection post-EK, and facilitate comparisons in graft survival in different experimental conditions.

In patients and animal models, graft rejection manifests as the opacification of the cornea [28,29]. The opacification of the corneal graft is attributed to immune cell infiltration, neovascularization, corneal thickening and irregularities, and the presence of edema [29]. In this study, we establish an objective scale for the precise assessment of graft failure, rejection, and acceptance, thereby enhancing our ability to monitor and manage graft outcomes in the EK model. Moreover, we delineate the immune mechanisms and elucidate their roles in graft rejection, which will allow us to develop therapies to improve the graft outcomes in EK.

## 2. Materials and Methods

### 2.1. Animals

We used 8–10-week-old male BALB/c mice as recipients and C57BL/6 mice as donors (Charles River Laboratories, Wilmington, MA, USA). All the animals used in the experiments were housed at the vivarium in the Schepens Eye Research Institute of Massachusetts Eye and Ear, in a pathogen-free environment. All experiments were approved by the Institutional Animal Care and Use Committee and were conducted in strict accordance with the Association for Research in Vision and Ophthalmology Statement for the Use of Animals in Ophthalmic and Vision Research.

### 2.2. Surgical Procedure

EK was performed as per the surgical protocol previously established by our group [24]. Briefly, for syngeneic EK, the endothelium, Descemet’s membrane, and posterior stroma were harvested from BALB/c mice and transplanted into BALB/c recipient mice. In allogeneic EK, the tissue was derived from C57BL/6 mice and transplanted into BALB/c recipients. Post-transplantation, the graft survival was assessed and graded using slit-lamp biomicroscopy and optical coherence tomography (OCT) for 16 weeks.

### 2.3. Donor Tissue Preparation

The donor C57BL/6 and BALB/c mice were euthanized using CO_2_ inhalation for allogeneic and syngeneic transplantation, respectively. We used a jeweler’s forceps to create counterpressure, and, subsequently, a partial thickness stromal incision was performed with 1.5 mm trephine. Next, we introduced a 30 G needle angled in a bevel-down position into the groove made by the trephine at the 5 o’clock position and we used the Vannas scissors and jeweler’s forceps to separate the anterior two-thirds of the stroma from the posterior one-third of the tissue. Thereafter, 50 μL of viscoelastic material was injected into the anterior chamber (AC) using a 30 G needle. Subsequently, the remaining posterior corneal tissue consisting of posterior stroma, Descemet membrane, and corneal endothelium was excised for transplantation.

### 2.4. Graft Implantation

BALB/c mice receiving transplants were anesthetized via the intraperitoneal injection of ketamine/xylazine, as approved in our protocol. The non-operated eye on the opposite side was shielded with GenTeal lubricant gel during the procedure. The central 2 mm of the recipient’s cornea was delineated with a light application of a trephine. Using a 30 G needle (bevel down), an incision tunnel was made in the trephine groove at the 3 o’clock position. Next, we injected 50 μL of viscoelastic material into the anterior chamber (AC) to maintain its depth. Subsequently, we introduced the tip of a bent 30 G needle into the AC, with the bevel facing upwards. Using a 2 mm trephine mark as a guide, we scrapped the Descemet’s membrane and mechanically removed the corneal endothelium. The EK donor tissue was gently introduced into the AC through the stromal tunnel, positioning it within the trephine mark. We injected 10μL of viscoelastic material to secure the placement of the donor tissue in proximity to the host’s stroma. The graft was centered within the stripped host cornea using the tip of the bent 30 G needle and viscoelastic material was removed from the AC, leaving the minimum amount necessary to promote graft adherence to the recipient corneal tissue. The stromal tunnel was closed using a single 11-0 nylon intrastromal suture. The post-procedure assessment of the transplanted corneal tissue included the measurement of AC depth, the integrity of the iris, the shape of the pupil, and suture placement. All the recipients were administered with a topical antibiotic ointment consisting of neomycin, bacitracin, and polymyxin B in the conjunctival sac. Finally, the animals were given a subcutaneous injection of buprenorphine HCl for analgesia.

## 3. Post-Operative Assessment

**Figure 1:**
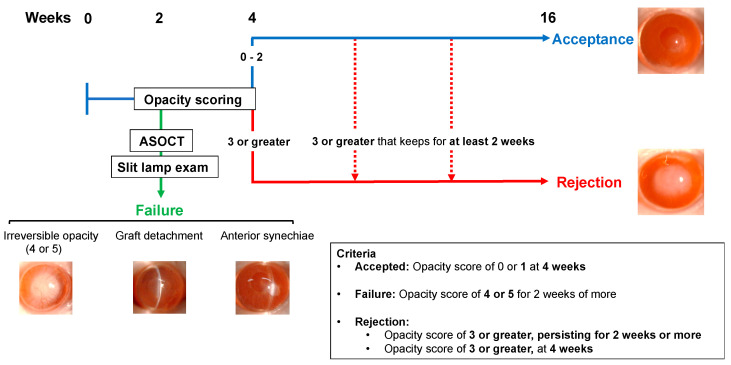
Algorithm for the clinical assessment of graft outcomes in a murine model of endothelial keratoplasty.

The recipient animals were assessed by slit-lamp examination and corneal opacity scoring, and anterior segment OCT was performed (Figure 1). The harvested corneas were immunostained to evaluate the corneal endothelial cell (CEnC) density in the graft recipients. Finally, in vitro assessment was performed to delineate the immune response in the different post-procedural outcomes.

### 3.1. Clinical Grading

**Figure 2:**
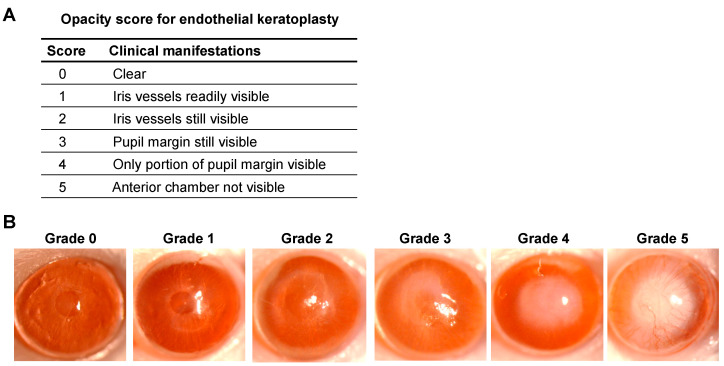
(**A**) Scoring system and (**B**) corresponding representative images for grading corneal opacity in a murine model of endothelial keratoplasty.

We developed a unique scoring system for grading EK outcomes, adapted from the scoring system previously used for a murine model of PK, by assessing the corneal opacity on slit-lamp examination [29]. The opacity in the transplanted cornea was graded (from 0 to 5) as follows (Figure 2):

- Grade 0—Clear: The cornea appears transparent and devoid of any opacities. Light is transmitted through the transplanted corneal tissue unimpeded, allowing clear visualization of intraocular structures.
- Grade 1—Iris vessels clearly visible: Mild corneal haze might be present; however, the blood vessels on the surface of the iris remain clearly visible through the transplanted corneal tissue.
- Grade 2—Iris vessels partially visible: A few of the vessels of the iris remain visible; however, they may appear less distinct or partially masked by corneal haze or superficial changes in corneal transparency.
- Grade 3—Pupil margin completely visible: The iris vasculature cannot be distinguished due to the opacity of the cornea; however, the margin of the pupil is completely visible.
- Grade 4—Pupil margin partially visible: The corneal opacity partially obscures the margin of the pupil. While portions of the pupil margin may still be visible, the opacity limits the clarity of its outline, resulting in partial visibility.
- Grade 5—Anterior chamber not visible: Severe corneal opacity is observed that completely obscures the anterior chamber of the eye. The dense opacity prevents any visualization of intraocular structures beyond the cornea, including the iris, pupil, and anterior chamber.

### 3.2. Optical Coherence Tomography

Along with slit-lamp examination, optical coherence tomography (OCT) was also performed [30]. As per the standardized procedure, mice were anesthetized with ketamine/xylazine, and topical anesthesia using a 0.5% Proparacaine ophthalmic solution (1–2 drops) was administered. To assess the status of the transplanted graft and central corneal thickness, we used an anterior segment–OCT system (Bioptigen Spectral Domain Ophthalmic Imaging System Envisu R2200) outfitted with a 12 mm telecentric cornea lens. Measurements were quantified utilizing the integrated software. 

### 3.3. Immunostaining

The corneas were harvested and subsequently fixed with 100% ethanol for 20 min and washed three times with PBS at 37 °C, as per the previously established protocol [31]. For permeabilization, the corneal tissue was incubated with 1% Triton X-100 (Sigma-Aldrich Inc., St. Louis, MO, USA) in PBS for 20 min at 37 °C. The harvested tissues were stained with a conjugated zonula occludens-1 (ZO-1) antibody in 1:200 (Thermo Fisher Scientific Inc., Waltham, MA, USA) overnight at 4 °C. Flat corneal mounts were placed onto glass slides with the grafted endothelium facing upwards. The CEnC density and morphology were assessed using confocal microscopy at ×200 magnification (Leica TCS-SP8; Leica GmbH, Germany). The acquired images were uploaded in Confoscan-4 software version 3.6.6. (NIDEK Technologies, Padua, Italy), and two authors manually performed endothelial cell assessment after delineating the cellular boundaries.

### 3.4. Enzyme-Linked Immunospot Assay

The enzyme-linked immunospot assay (ELISPOT) was performed for the quantification of directly and indirectly activated T cells, as per the established protocols [32]. Briefly, 96-well ELISPOT plates (Whatman Polyfiltronics, Newton, MA, USA) were first coated with the primary anti-IFN-γ (4 μg/mL) monoclonal antibody (BD Pharmingen, Franklin Lakes, NJ, USA) in sterile PBS for 48 h. Subsequently, the plates were washed, and antibody blocking was performed with 1% bovine serum albumin-supplemented PBS for 1.5 h. Next, we isolated purified T cells (5 × 10^5^ cells) by CD90.2-positive magnetic-activated cell sorting (MACS) from the draining lymph nodes (dLNs) of grafted BALB/c mice (6 mice/group, at 2 weeks post-transplantation of C57BL/6 donor corneas) and pooled them. The isolated T cells were incubated with C57BL/6 antigen-presenting cells (APCs; 5 × 10^5^ CD90.2-negative cells were sorted with MACS from spleen) for 48 h and the frequencies of directly allosensitized T cells were assessed.

To quantify the frequencies of indirectly allosensitized T cells, syngeneic BALB/c APCs (5 × 10^5^ cells) were pulsed with sonicated donor antigen (2 × 10^7^ APCs/mL derived from C57BL/6), followed by incubation with T cells harvested from dLNs of BALB/c recipients, with healthy C57BL/6 donor corneas used as controls. Subsequently, the cells were incubated with biotinylated anti-IFN-γ detection monoclonal antibody (2 μg/mL, BD Pharmingen) for 2 h at 37 °C. Subsequently, the cells were incubated with avidin-horseradish peroxidase (HRP) for 1.5 h and developed using a 3-amino-9-ethylcarbazole substrate for 30 min (MN 51-2577KC, BD Biosciences, Woburn, MA, USA). The resulting spots were analyzed with an ELISPOT image analyzer (Cellular Technology Ltd., Cleveland, OH, USA).

### 3.5. Statistical Analysis

Statistical analysis was performed using Prism for Mac (v.10.1.1; GraphPad Software Inc., La Jolla, CA, USA). We performed one-way analysis of variance (ANOVA) with Bonferroni correction to compare the means of three or more independent groups. The results are presented as mean ± SD. We performed simple linear regression analysis to assess the effect of CEnC loss on corneal thickness. We also performed a similar correlation analysis to evaluate the effect of T cell sensitization (in dLN) on CEnC density. Kaplan–Meier survival analysis was performed to compare the survival of allogeneic and syngeneic EK. The differences were considered statistically significant was considered when *p* < 0.05. 

## 4. Results

**Figure 3:**
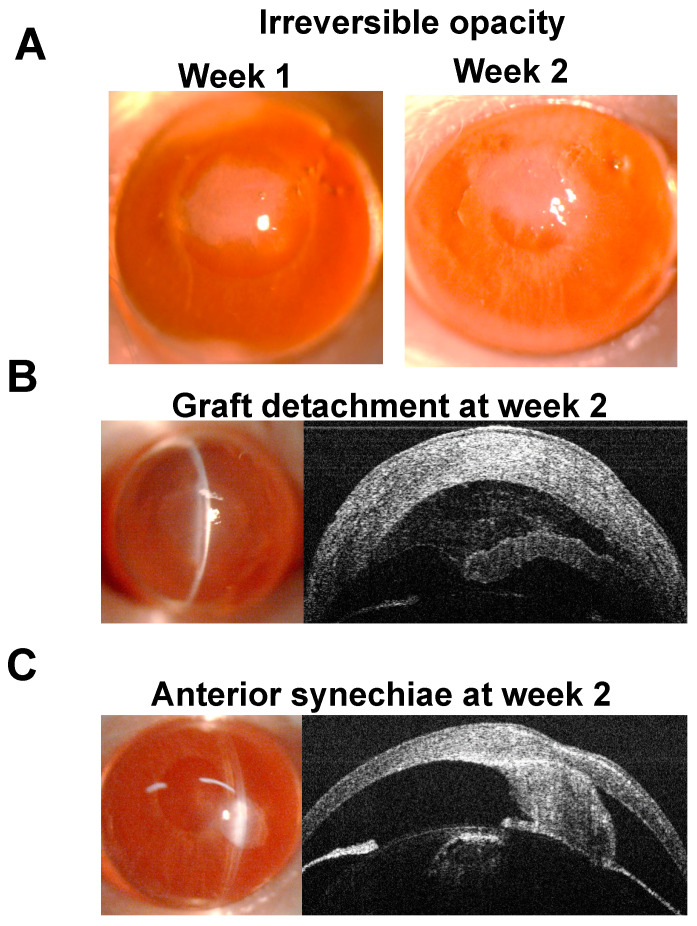
Representative slit-lamp examination and optical coherence tomography images of (**A**) irreversible opacity, (**B**) graft detachment, and (**C**) anterior synechiae, which are classified as graft failure in mice following endothelial keratoplasty.

The standardized and comprehensive clinical protocol for assessing the corneal graft following the endothelial keratoplasty (EK) is summarized in Figure 1. We performed the EK as per the established protocol and performed slit-lamp examination-based opacity scoring (Figure 2) and AS-OCT, weekly, for 16 weeks post-procedure. At week 2, two animals with irreversible opacity (grades 4 or 5), one animal with graft detachment and one animal with anterior synechiae were considered as graft failures and excluded from further assessment (Figure 3). At four weeks post-procedure, the animals with opacity scores of 3 or greater were considered rejected, indicating dysfunction of the grafted tissue. The animals with opacity scores of 0 to 2 were considered accepted at four weeks. However, if the opacity score worsened to 3 or greater for more than 2 weeks in the animals classified as “accepted” initially, these grafts were considered rejected as well.

**Figure 4:**
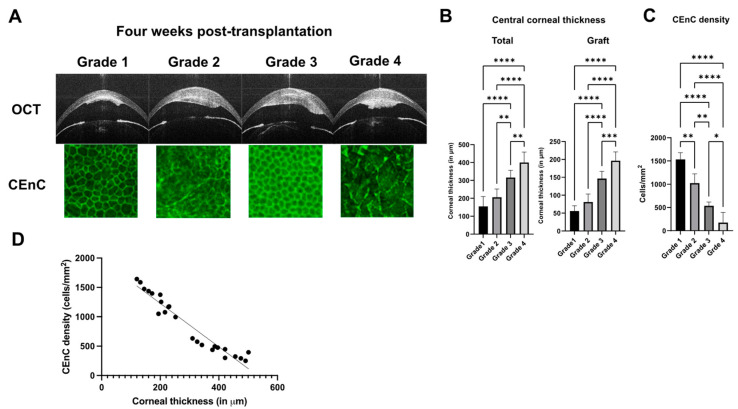
(**A**) Optical coherence tomography and corresponding zonula occludens-1 confocal images assessing the (**B**) total and graft corneal thickness, and (**C**) corneal endothelial cell density (CEnC), respectively. (**D**) Linear regression showed an inverse correlation between endothelial cell density and thickness in tissues with different grades of corneal opacity. The intergroup comparison was performed using a one-way analysis of variance test (* *p* < 0.05, ** *p* < 0.01, ***p < 0.001, **** *p* < 0.0001). Scale = 100 μm (for confocal imaging).

The 4-week time point post-transplantation is a critical juncture for assessing graft outcomes in the model. At this time point, all mice were stratified into distinct groups based on their opacity scores, which served as surrogate markers for the condition of the graft. Subsequently, we performed a comprehensive evaluation including OCT and immunostaining with ZO-1 to assess central corneal thickness and endothelial cell density in 4–6 animals per group. We observed that the total corneal and graft thickness corresponded to the corneal opacity grades and increased progressively from grades 1 to 4 (Figure 4A,B). Increased corneal thickness in higher grades of corneal opacity is indicative of progressive tissue remodeling and graft edema. Moreover, CEnC density exhibited a gradual decline corresponding to worse opacity scores, suggesting concomitant loss or the dysfunction of these cells with worsening graft condition (Figure 4C). On performing linear regression, we observed an inverse correlation between CEnC density and corneal thickness (*p* < 0.0001) (Figure 4D). These results indicate that the loss of CEnC in corneas with higher grades of corneal opacity resulted in the loss of barrier function, subsequent corneal edema, and, eventually, graft failure.

**Figure 5:**
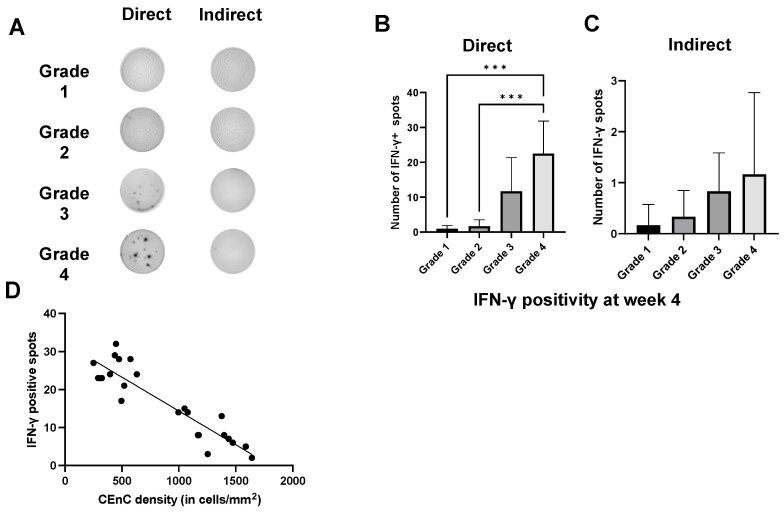
(**A**) The enzyme-linked immunospot (ELISPOT) assay showed a significant association between increased (**B**) direct allosensitization of Th1 cells (indicated by higher IFN-γ^+^ spots at four weeks) with a higher grade of corneal opacity in recipients following allogeneic EK. (**C**) A moderately higher indirect Th1 allosensitization was observed in animals with a higher grade of corneal opacity following allogeneic EK. (**D**) Linear regression analysis showed an inverse correlation between IFN-γ^+^ spots (direct pathway) on ELISPOT assay and CEnC density in tissues derived from mice with various grades of corneal opacity. Intergroup comparison was performed using a one-way analysis of variance test (*** *p* < 0.001).

Next, we quantified the alloreactive T cell frequencies using the ELISPOT assay (Figure 5A). We observed significantly higher frequencies of direct pathway-sensitized T cells in grade 4 compared to grade 1 and 2 animals, whereas the frequencies of these cells were only moderately higher compared to those of grade 3 mice (Figure 5B). The indirect pathway-sensitized T cell frequencies only increased moderately with worsening corneal opacity grading (Figure 5C). This finding suggests a heightened immune response in corneal grafts characterized by severe opacity. Importantly, the direct and indirect IFN-γ positivity was nearly undetectable in the grade 1 and 2 groups, indicating minimal Th1 cell activation in these cases. On performing linear regression, we observed that the higher direct IFN-γ positivity resulted in significantly lower CEnC density (*p* < 0.0001) (Figure 5D). These observations indicate the deleterious effect of Th1 cells on CEnCs, eventually leading to graft rejection in allogeneic recipients.

**Figure 6:**
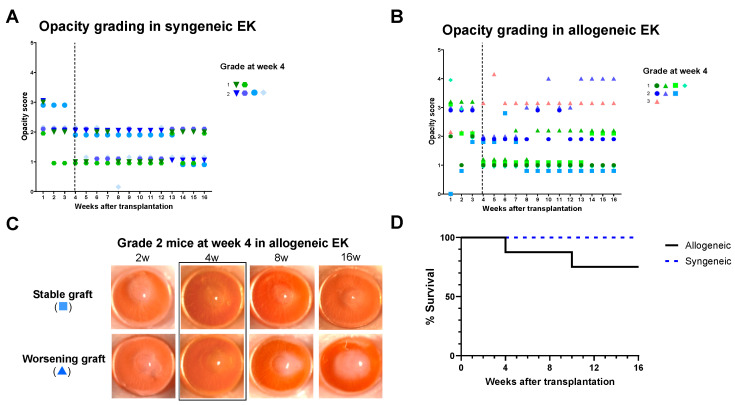
Corneal opacity grading in (**A**) syngeneic and (**B**) allogeneic EK over 16-week post procedure. At four weeks post-procedure, recipients with syngeneic EK remained consistently low corneal opacity scores, (**C**) whereas the animals with allogeneic EK had higher scores which were variable during the remaining 12 weeks of follow-up. (**D**) The Kaplan–Meier curve shows that all the recipients with syngeneic EK showed graft survival at the end of 16 weeks, whereas the graft survival rate was 75% in allogeneic EK recipients.

In the final analysis, eight animals with allogeneic EK and six animals with syngeneic EK were included. On comparing the outcomes of allogeneic and syngeneic EK, we observed a clear distinction in the opacity scores over time. The opacity scores in syngeneic EK remained consistently low and between grades 0 and 2 (Figure 6A). However, in allogeneic EK, despite low opacity scores at the 4-week time point, the outcomes of the grafts were highly variable, with some grafts having a stable opacity score and other grafts having a worsening opacity over time (Figure 6B,C). Kaplan–Meier analysis of corneal grafts showed indefinite graft survival in all the animals that underwent syngeneic EK (Figure 6D). However, animals that underwent allogeneic EK had a lower graft survival beginning at the 4-week time point, and a 25% graft rejection rate at the 16-week study end point.

## 5. Discussion

In this study, we performed an extensive assessment of a well-established murine model of EK [24] to develop a rigorous grading system for post-procedure evaluation, facilitating the precise characterization of graft outcomes. Furthermore, our investigation unveiled a significant association between graft rejection and heightened T cell activation, which was particularly pronounced in allogeneic transplantation settings. This observation underscores the pivotal role of immune-mediated processes in driving endothelial graft rejection, a phenomenon that is notably less frequent in syngeneic transplants. These findings offer insights into the immune mechanisms governing outcomes post-EK, and allow for the development of targeted therapeutic strategies aimed at enhancing graft survival and improving clinical outcomes.

EK is a minimally invasive procedure compared to PK, thus significantly reducing the risk of graft rejection [13,15]. Moreover, lower frequencies of donor antigen-presenting cells and a lesser amount of immunogenic tissue are observed in EK. The minimal use of suturing and mild postoperative inflammation mitigates inflammatory responses in graft sites [33,34]. Despite the lower immunogenicity inherent in EK, it remains imperative to develop insights pertaining to the underlying immunological processes that induce graft rejection [35].

Although cat- and rabbit-based models for EK have been developed previously, the utilization of a murine model is particularly advantageous due to the easy availability of murine-specific reagents, recombinant factors, neutralizing antibodies, and transgenic strains that can be harnessed for investigative purposes [26,36]. This is particularly significant as murine strains with well-defined immunogenetics offer a unique opportunity for the in-depth exploration of immune processes, unlike outbred animals. Previously, murine models have been instrumental in elucidating the pathophysiology of numerous human autoimmune diseases and various forms of organ transplantation [37,38]. 

Immune-mediated rejection following corneal transplantation involves a complex interplay of various immune cells and cytokines. Among these, IFNγ-producing CD4^+^ Th1 cells play a pivotal role as the primary effector cells in graft rejection [39]. Th1 cells orchestrate a cascade of inflammatory responses inducing tissue damage and graft failure. The ability of these cells to stimulate macrophages, recruit other immune cells, and induce cytotoxicity is central to the process of graft rejection [40]. Furthermore, Th1 cells are known to promote the production of pro-inflammatory cytokines and chemokines, amplifying the immune response against the graft. 

In this study, we established a protocol for assessing graft outcomes in mice following EK. To assess the outcomes of EK, we performed weekly examination for 16 weeks to detect the changes in corneal opacity, specifically in animals that underwent allogeneic EK. In animals that underwent allogeneic EK, the 4-week time point was critical as the opacity scores were highly variable during the follow-up period. 

Due to the high variability in the clinical outcomes in allogeneic EK, we further investigated the immunological mechanisms underlying graft rejection. We evaluated the association between the direct and indirect allosensitization of Th1 cells (using ELISPOT assay) and the degree of corneal opacity. We observed a heightened direct Th1-mediated immune response in corneal grafts with severe opacity. This association highlights the potential application of IFN-γ (expressed by Th1 cells) as a quantitative marker for graft rejection in EK. Moreover, these results provide valuable insights into the immunological mechanisms underlying corneal graft rejection in EK, and the potential development of efficacious therapeutic interventions to mitigate rejection and improve the outcomes of EK. The development and standardization of clinical assessment in a murine model for EK holds promise in terms of advancing our understanding of the immunological mechanisms underlying this procedure.

The anatomy of the mouse eye is a major challenge in assessing the mechanisms of graft rejection. The shallow anterior chamber increases the risk of damage to the corneal endothelial cells due to manipulation during the surgical procedure [41]. Additionally, in the murine model of EK, the graft is placed with forceps compared to an insertion device or cartridge in humans, which can also lead to increased endothelial cell loss [42].

## 6. Conclusions

In conclusion, our study successfully establishes an accurate assessment protocol for use detecting corneal rejection in a murine model of EK. Furthermore, we demonstrated the role of Th1 cells in immune-mediated graft rejection in allogeneic EK. This model has promise as a valuable tool for further investigations into the mechanisms of rejection in endothelial keratoplasty.

## Data Availability

Data are available from the corresponding author upon request.
